# Embracing imperfection: Carbon offset markets must learn to mitigate the risk of overcrediting

**DOI:** 10.1093/pnasnexus/pgaf091

**Published:** 2025-03-15

**Authors:** Bodie Cabiyo, Christopher B Field

**Affiliations:** Stanford Woods Institute for the Environment, Stanford University, 450 Jane Stanford Way, Stanford, CA 94305, USA; Stanford Woods Institute for the Environment, Stanford University, 450 Jane Stanford Way, Stanford, CA 94305, USA

## Abstract

The role of carbon offset markets in accelerating climate action has been widely anticipated. Recently, fundamental questions have emerged about the role of carbon credits in the wake of widespread quality critiques of existing carbon offset projects. In tandem, many large corporate buyers are slowing their voluntary investments in carbon credits or shifting to less public vehicles for those investments. The discourse to date has focused on raising the quality bar of carbon credits, which is critical. However, it has broadly overlooked the fact that heterogeneous quality and uncertainty may be an inherent attribute of carbon projects. Carbon credits are an intangible good that may always have imperfect accounting. The success of carbon offset markets in part depends on embracing this imperfection, rather than obscuring or ignoring it. Thus, we propose that carbon offset markets need new mechanisms that account for—and encourage—iterative methodological improvements in how carbon credits are issued. These may include a variety of insurance-like mechanisms, warranties, and ex post adjustment mechanisms. They each work to guarantee that issued credits deliver on their promises, much like how carbon storage reversals are already guaranteed by credit registries. Market actors have already started testing such solutions, from true insurance products to buffer pools to discounted credit portfolios. Here, we highlight opportunities and challenges to achieve this goal, including the potential role of carbon registries, private actors, and large institutions.

## Avoided deforestation as a case study

Overcrediting—issuing more credits than can be conservatively justified by real mitigation activity—is at the heart of many critiques of the voluntary carbon market (VCM). The evolution of Reducing Emissions from Deforestation and Degradation (REDD), in particular, is instructive for the broader market. REDD standards and approaches have been continually revised in response to 17 years of public discourse and scientific progress (1). However, this process of revision has led to uneven results. On one hand, individual REDD projects have been repeatedly shown to rapidly reduce emissions at costs as low as USD$1/ton CO_2_ (2–5). On the other hand, in the VCM, studies have found that as much as 90% of REDD credits issued by Verra failed to deliver their stated reductions (6, 7). Similarly, third-party ratings agencies have recently arisen to vet the quality of VCM credits and have identified deep, systematic flaws across many project types, including REDD (8, 9). REDD projects have been repeatedly proven to perform, but the VCM mechanisms used to verify those projects are broken. Due to its long history, high potential, and high-profile critiques, REDD may be a “canary in the coal mine” for carbon offset markets more broadly: emerging carbon credit pathways may yet encounter the same challenges. These hard-won lessons can inform the growth and success of new areas of VCM growth, including the burgeoning market for carbon dioxide removal credits.

At the same time, new approaches to crediting mark a meaningful jump forward in assuring quality. Again, REDD is an instructive example. Jurisdictional-scale REDD crediting, for example, is rapidly expanding and represents clear improvement over past project-based approaches, including through reduced market leakage and straightforward baselines (5, 10). Baselines—the counterfactual comparison against which credits are generated—provide insight into this trend. Under the new ART-TREES standard, for example, baselines are determined through a jurisdiction-wide lookback period of 5 years (11). This approach eliminates the heterogeneity and opacity in baseline setting inherent to previous project-scale REDD approaches, significantly diminishing perverse incentives. Outside of REDD, new forest methodologies are emerging, such as the new Verra Afforestation, Reforestation, and Revegetation (ARR) methodology, that promise greater certainty in setting dynamic, empirically verified baselines and constraining other factors like market leakage (12). Such methodological advances are based on an evolving understanding of the science and practice of carbon credits. They represent meaningful improvement in quality over the methodologies that preceded them.

Still, these new approaches are untested and are likely flawed in important ways. The science of carbon measurement is far from perfect, and even state-of-the-art measurement tools have error bars on their estimates. As quantification of carbon credits improves, even the best quantification today may still be invalidated by the science of tomorrow. This raises a critical question: how can carbon offset markets be structured so that learning is expected and climate progress is not encumbered by that learning?

## The VCM as it stands

The VCM is small and heterogeneous compared with markets for other assets (13). Compliance carbon markets like the European Trading System operate at a larger scale, but they are still small compared with the scale of markets promised by Article 6 and projections for carbon dioxide removal needed to reach global climate targets (14, 15). Despite overall growth, distrust in credit quality is a defining feature of the VCM today (16).

Several layers of quality assurance have emerged to mitigate risk in credit procurement. Registries work to mitigate risk through open methodology development, but project developers often influence the process by writing the methodologies and participating in stakeholder comment periods^a^. In most methodologies, projects must take an “uncertainty deduction,” with no chance for recouping deducted credits if uncertainty is reduced over time. Third-party verifiers work to ensure that methodologies are followed prior to credit issuance. Standards bodies like the Integrity Council for Voluntary Carbon Markets and Carbon Offsetting and Reduction Scheme for International Aviation aim to raise the quality floor by creating minimum requirements for methodologies to receive their label (17, 18). Ratings agencies, which have only existed since 2020, vet credits postissuance (8, 9). Finally, buyers often conduct bespoke project-level diligence in addition to the preceding third-party quality assurance (19). All told, a given credit may pass through 4 or more levels of quality assurance from preissuance to retirement. This overlapping risk mitigation exists in part because issued credits are much more likely to underperform than overperform. Overcrediting is common and undercrediting is rare (7, 16, 20, 21).

Overcrediting is a key risk factor in the production and use of carbon credits. To date, the question of who should bear the risk of overcrediting has been implicit in the architecture of carbon offset markets. By default, there is no widespread recourse for overcrediting, so the risk is born by the climate itself. Overcrediting almost inevitably leads to higher net emissions than were promised by the offsetting system. Because offsetting is arithmetically a substitution of a ton emitted for a ton credited, a carbon credit that does not deliver on its label results in higher net emissions via imperfect substitution (22). Buyers also bear the reputational risk associated with using faulty credits. In practice, some buyers have tried to mitigate this risk through extensive prepurchase diligence, by not making public claims, or by overbuying credits where they see potential for poor quality (23). For example, some sustainability executives have reported reticence to publicly disclose carbon credit purchases to avoid public invalidation of those purchases, a phenomenon called “green hushing” (23, 24). These firms often see greater reputational downside than upside in making environmental claims (25).

Structural conditions in the market today foster systematic overcrediting and undermine its credibility. Because offsets represent an intangible product—an emission reduction or removal that cannot be easily verified—they are inherently susceptible to subjective accounting choices and methodological loopholes (20, 21). This opacity often interacts with principal-agent dynamics, in which the project developers and other intermediaries who issue the credits do not share the same incentives as the ultimate buyers (26). Often, each actor in the supply chain of such a good is financially incentivized to maximize the volume of credits, rather than their veracity (27, 28). These conditions create a fertile environment for consistent bias, in which market mechanisms fail to disincentivize overcrediting (29, 30). Without structural reforms—ones that realign incentives, enhance transparency, and tighten accountability—the market for offsets will remain prone to fundamental flaws that no financial instrument, underwriting process, or risk-hedging strategy can fully overcome. Such structural reforms have been successfully executed in related markets (22).

## Physical reversals as an analogue for overcrediting

Physical carbon reversals may provide a useful analogue to the problem of overcrediting. When stored carbon is physically released to the atmosphere through, for example, a wildfire, it can be deemed a “reversal event” by the registry, and the released carbon is replaced by credits from the registry buffer pool. The buffer pool, an insurance-like mechanism, is a large pool of credits that can be retired in the event of reversal events. It works best if the pool is diversified across projects and types of risk (31, 32). Credits are contributed to the buffer pool from all projects when credits are issued. For example, Verra operates a buffer pool of over 70 million tons, accrued through contributions of roughly 10% to 20% of issued credits from many project types (21). Buffer pools, although imperfect, are a powerful mechanism because they can diversify risk across many carbon projects and pay out in carbon credits (rather than, for example, dollars, like a normal insurance policy).

There is no buffer pool for other aspects of credit quality, beyond physical reversal. When credits are proven to be miscalculated, the claimed climate benefits are functionally reversed, but it is a reversal not protected by buffer pools or any other insurance mechanism. Unlike physical reversal, the risk of overcrediting is only partially covered in the carbon offset market ecosystem. The result is that buyers of credits—and the climate—may face negative impacts. Ensuring against physical reversals is simultaneously essential and insufficient: credits should be protected against other reversals of claimed benefits.

## Exploring pathways to buffer overcrediting risk

Multiple types of institutions can play and are actively playing a role in reducing overcrediting risk. Carbon offset markets already involve a diverse ecosystem of actors to perform various checks and balances. A mix of new solutions may ultimately create the mosaic of underwriting necessary to adequately buffer against overcrediting risks. Importantly, these solutions are best suited to mitigate uncertainty and risk, rather than deep systematic bias, in crediting. Inaccuracy in crediting methodologies becomes deep systematic bias when it accounting errors are large and one-directional, like when loopholes and optimistic assumptions enable pervasive overcrediting by market actors. Consistent bias has been prevalent in the methodologies historically used for credit types like REDD and renewable energy, leading to outcomes in which most credits are either nonadditional or greatly miscalculated (7, 20). A market oversupplied with “lemons”—faulty credits, here—is likely to fail due to lack of buyer confidence (33, 34). Deep systematic bias must be addressed first before risk mitigation approaches can be fully effective.

### Registries

Carbon registries already play a central role in protecting against physical reversals through the buffer pool. Similar buffer mechanisms could also mitigate the risk of overcrediting. In the simplest form, registries would expand the definition of what constitutes a “reversal” to include overcrediting events. This functions as a warranty on uncertain carbon accounting (16). An expanded definition of reversals likely necessitates 2 key updates to the existing system for addressing reversals: ex ante (before issuance) measurement of uncertainty and ex post (after issuance) monitoring of overcrediting.

**Fig. 1.**
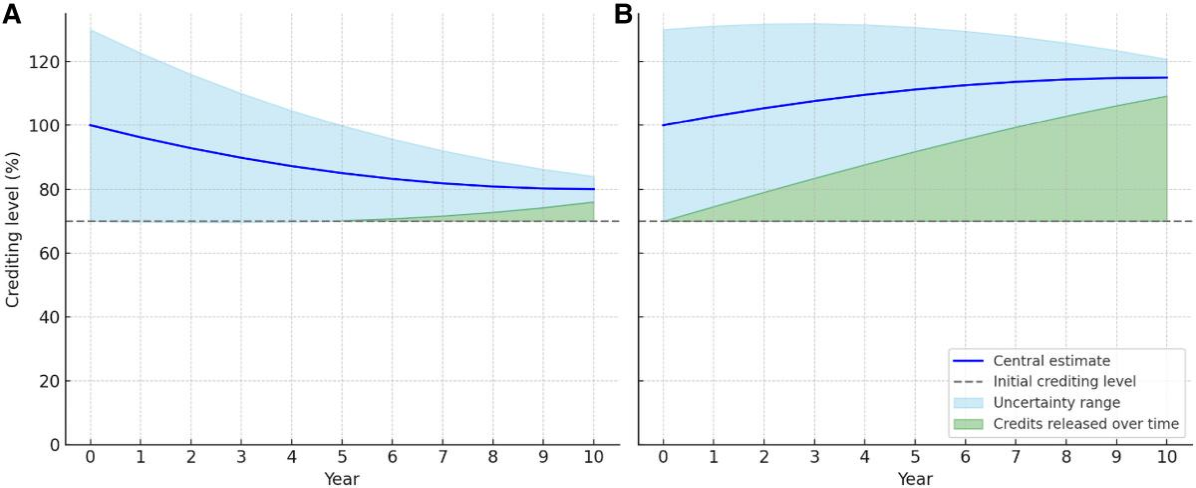
Schematic representation of approaches to buffering carbon credit uncertainty. A crediting level of 100% is reflective of how credits would be calculated using existing methods (i.e. using the central estimate today to calculate credit volumes), which could produce either overcrediting (A) or undercrediting (B) over time. Today, overcrediting is ubiquitous and undercrediting is rare. The “initial crediting level” represents a novel conservative approach that releases credits as uncertainty diminishes over time.

First, a revised allocation of credits to the buffer pool would be based on not just physical reversal risk, but also the risk of a future invalidation of credit calculations. Registries may choose to maintain this pool separately from the existing buffer pool for physical reversals. The risk of overcrediting would be estimated based on both known and unknown uncertainties in the methodology. Conservatively, this buffer allocation would be based on the lower bound of uncertainty (Figure 1). In some cases, high uncertainty may mean very few credits are generated initially. Most standards already include an “uncertainty deduction,” often taken as a flat deduction from total credits generated. The uncertainty deduction is often 10% or less and may vary slightly depending on project characteristics (11, 12, 21). This offers a simple model for an uncertainty buffer but is fundamentally different in that (i) it may inadequately represent uncertainty and (ii) credits are not later released for good performance.

Second, registries would need to develop a robust way to measure overcrediting. Likely the simplest approach to measurement is to define crediting invalidation via methodological updates: invalidated credits are those that would not be generated under the newest methodology rules. Such a system would insure credit quality against advances in carbon calculations, much in the same way that credits are insured against physical reversals. Unlike physical reversals, it could also insure against undercrediting (i.e. cases in which a default value underestimates the true carbon benefits of a project) (Figure 1B). Today, undercrediting is rare and is unrewarded by registry mechanisms. Critically, such a system would introduce both downside risk and upside risk to project developers, potentially reducing their ability to anticipate credit generation—and revenue—into the future. Speculation in credit generation would be a core element of investment into these projects.

A key feature—or perhaps a bug—of the current system is what might be described as vertical integration in the governance of carbon credits. Here, vertical integration refers to a single entity, a registry, managing multiple stages of the credit supply chain, including methodology updates, credit issuance, and adjustments for reversals. While this integration offers efficiency, it may also create conflicts of interest. Today, most registries generate revenue by charging a fee for each credit issued or retired, which may create a disincentive to reduce credit volumes (27). For example, if a given methodology update would reduce total credits issued, a registry may have a financial incentive to avoid that update. Conflicts of interest due to registry vertical integration may be diametrically opposed to continuously addressing overcrediting. New independent, science-led, nonprofit standard writing bodies are testing the potential to reduce vertical integration in the standard setting, credit registry process (35, 36).

### Private sector

Private sector innovation has recently yielded multiple promising solutions to overcrediting. Insurance companies can play a role in providing a financial guarantee of credit quality. For example, insurance company Swiss Re highlighted the potential role of insurance companies in carbon offset markets but also emphasized several key hurdles to participation, including the long time horizons and high uncertainty associated with insuring credits (37). Start-up insurance brokers are working to address these challenges, but to date have mostly focused on insuring credit delivery risk for ex ante credits, not on credit quality (38).

Like insurance companies, carbon credit brokers are beginning to play a role in protecting against overcrediting through ex post adjustments of credit volumes. For example, credit brokers have recently piloted solutions to overcrediting by internally recalculating—or “rightsizing”—credits for each project in a portfolio (39, 40). In the case of REDD, this takes the form of recalculating the baseline. With projects like landfill gas capture or improved cookstoves, it takes the form of adjusting carbon accounting downward to match more conservative accounting assumptions. Critically, rightsizing credits is an approach that works well to address errors in accounting; these approaches are ill-suited to address additionality or permanence issues in individual projects. Although this approach is novel, its success hinges on the transparency and accuracy of how credits are recalculated. Presently, there is no independent audit mechanism built into such systems. Such solutions are promising, but they function best at large scales adequate to diversify risks across many unrelated carbon projects.

### Large institutions

Large institutions, such as governments and international financial institutions like the World Bank, may leverage their size and influence to serve a role in mitigating overcrediting risk. While these institutions have expressed broad interest regulating voluntary carbon markets, they have played a relatively minor role to date. Governments and international financial institutions may rely on traditional insurance tools to protect against overcrediting. For example, in 2024, the World Bank's Multilateral Investment Guarantee Agency launched a new initiative to de-risk carbon markets by providing guarantees that address political and regulatory risks (41). This platform aims to encourage private investment in carbon markets by reducing the uncertainty associated with government interventions, such as policy changes or export restrictions on carbon credits, as has occurred or is expected to occur in India, Zimbabwe, and other countries (42, 43). Expanding this model, insurance or guarantee mechanisms could be explored for overcrediting risk, in which miscalculated credits would be viewed as a breach of contract with credit buyers. Despite government announcements indicating broad support for the VCM, like those from the White House, large institutional actors have as of yet played a minor role in defining VCM architecture (44). Any involvement by large institutions should complement existing market-based mechanisms and focus on areas where private solutions are less viable. Lessons from past financial crises underscore the need for transparent, bounded guarantees to prevent systemic risks or unintended societal impacts (45).

## Conclusion

The ongoing challenges tied to overcrediting of carbon are real and substantial, and the repercussions are largely borne by the climate itself. While current measures like buffer pools and buyer diligence provide partial solutions, they remain insufficient and limited in scope. A substantial shift in the existing models of addressing overcrediting may be necessary to uphold the enduring credibility of carbon offset markets. Registries, the private sector, and large institutions all may play independent roles in addressing overcrediting risk. But ultimately, these solutions are likely to work best in concert as a mosaic of risk underwriting and regulation. The expanded role of registry buffer pools and private-sector innovation could prove collectively significant in confronting this challenge. Addressing the questions that lie ahead is crucial as the landscape of carbon offset markets evolves:

1. What incentive structures will emerge from approaches to reversing overcrediting, and how might they influence behavior?
2. How can approaches to reduce overcrediting strike a balance to avoid placing excessive risk on project developers and ensure long-term trust in the crediting systems?
3. How can overcrediting risk mitigation be designed to reward prudence and reduce perverse incentives across the carbon credit value chain?

In the context of rapidly evolving and growing carbon offset markets, proactive, innovative, and collaborative efforts are indispensable in forging resilient, transparent market structures that genuinely contribute to combatting climate change. Mitigating the risk of overcrediting may be a key to realizing the potential of this essential climate mechanism.

## Data Availability

No data were used in this study.

## Notes

a For example, the American Carbon Registry's recent methodology, Improved Forest Management On Non-Federal U.S. Forestlands 2.1, is co-written by two large project developers, Anew and Finite Carbon, both of which have been identified as bad actors by media and academics.
